# Burnout among Portuguese healthcare workers during the COVID-19 pandemic

**DOI:** 10.1186/s12889-020-09980-z

**Published:** 2020-12-07

**Authors:** Ivone Duarte, Andreia Teixeira, Luísa Castro, Sílvia Marina, Carla Ribeiro, Cristina Jácome, Vera Martins, Inês Ribeiro-Vaz, Hugo Celso Pinheiro, Andreia Rodrigues Silva, Miguel Ricou, Bruno Sousa, Cristiana Alves, Andreia Oliveira, Paula Silva, Rui Nunes, Carla Serrão

**Affiliations:** 1 MEDCIDS - Department of Community Medicine, Information and Health Decision Sciences, University of Porto, Porto, Portugal; 2 CINTESIS - Center for Health Technology and Services Research, Faculty of Medicine, University of Porto, Porto, Portugal; 3 School of Health of Polytechnic of Porto, Porto, Portugal; 4 Pulmonology Department, Centro Hospitalar de Vila Nova de Gaia/Espinho, Vila Nova de Gaia, Portugal; 5 Porto Pharmacovigilance Centre, Faculty of Medicine, University of Porto, Porto, Portugal; 6 Department of Internal Medicine, Tâmega e Sousa Hospital Center, Penafiel, Porto, Portugal; 7 Darque Health Care Center, Alto Minho Local Healtn Care Center, Viana do Castelo, Portugal; 8 CBIOS – Research Center for Biosciences and Health Technologies, Lisboa, Portugal and Health Service of Autonomous Region of Madeira, Funchal, Portugal; 9 Portuguese Institute of Oncology of Porto, Porto, Portugal; 10 School of Education of Polytechnic of Porto, Centre for Research and Innovation in Education (inED), Porto, Portugal

**Keywords:** COVID-19, Healthcare workers, Burnout; Stress, Depression, Resilience, Life satisfaction

## Abstract

**Background:**

During COVID-19 pandemic, healthcare workers (HCWs) have had high workload and have been exposed to multiple psychosocial stressors. The aim of this study was to evaluate HCWs in terms of the relative contributions of socio-demographic and mental health variables on three burnout dimensions: personal, work-related, and client-related burnout.

**Methods:**

A cross-sectional study was performed using an online questionnaire spread via social networks. A snowball technique supported by health care institutions and professional organizations was applied.

**Results:**

A total of 2008 subjects completed the survey. Gender, parental status, marriage status, and salary reduction were found to be significant factors for personal burnout. Health problems and direct contact with infected people were significantly associated with more susceptibility to high personal and work-related burnout. Frontline working positions were associated with all three dimensions. Higher levels of stress and depression in HCWs were significantly associated with increased levels of all burnout dimensions. Higher levels of satisfaction with life and resilience were significantly associated with lower levels of all burnout dimensions.

**Conclusions:**

All three burnout dimensions were associated with a specific set of covariates. Consideration of these three dimensions is important when designing future burnout prevention programs for HCWs.

**Supplementary Information:**

The online version contains supplementary material available at 10.1186/s12889-020-09980-z.

## Background

The impact of the COVID-19 pandemic on healthcare workers (HCWs) has been tremendous. The impact is not only related to increased workload, but also fear of the disease being contracted by themselves and their families, working with new and frequently changing protocols, limited personal protective equipment, caring for patients who are very sick and quickly deteriorating, and caring for colleagues who have also fallen ill [1–4]. This pandemic has exacerbated stressors in healthcare systems, in which HCWs burnout in response to workplace stress is already an epidemic [5].

According to recent studies, some HCWs have developed psychological distress [6, 7], fatigue and burnout [8], while facing the COVID-19 pandemic. The knowledge on the impact of COVID-19 on HCWs’ mental health is still incipient. However, some insights on possible mental health consequences of severe infection outbreaks may be obtained from studies conducted in the settings of other outbreaks, such as Severe Acute Respiratory Syndrome (SARS) in 2003 and Middle East Respiratory Syndrome (MERS) in 2012. These studies indicate that physicians have experienced adverse psychological disorders such as anxiety, fear, and stigmatization during and after past outbreaks [9–11].

HCWs not only have a high exposure risk to infection, but also a high burden of mental health stress, especially those working directly with people who have or are suspected of having COVID-19 [12]. Lai et al. [6] conducted a study in China with 1257 HCWs (60.8% nurses and 39.2% physicians), of which 41.5% were frontline professionals. They concluded that 71.5% suffered from distress, 50.4% suffered from symptoms of depression, and 44.6% suffered from anxiety, and these consequences were more evident in female nurses. Another study from China with 134 frontline workers (41% nurses, 35.1% physicians, 23.9% support staff) showed that more than half of the HCWs had moderate to severe levels of stress perception.

Depressive and anxiety symptoms are more common among women [13]. In Italy, Rossi et al. [14] concluded that 24.7% of HCWs had symptoms of depression, 21.9% had high perceived stress, and 19.8% had anxiety symptoms. Another study conducted in Italy shows that anxiety was reported by 16,16% and depression by 20,3% of HCWs [15]. These studies help to understand the psychosocial impact of COVID-19 on HCWs, but to our knowledge, none of them explored the exposure to other recurrent stress factors that can lead to burnout [16].

A possible definition to burnout is a “*state of physical, emotional and mental exhaustion that results from long-term involvement in work situations that are emotionally demanding”* [17]. Burnout is experienced by the person with high level of physical, emotional and psychological fatigue [18]. In support of an integrative view of health, Kristensen et al. [17] indicate that the core of burnout is fatigue and exhaustion, defined by the dimensions of person burnout, work-related burnout and client-related burnout. Burnout has been considered a very relevant occupational health hazard among HCWs. Individuals with burnout demonstrate a reduction in professional performance, greater probability of medical error, higher rates of absenteeism, lower commitment to the job and the employer, lower job satisfaction, higher occurrence of medical leave, and greater personal suffering [19]. Also burnout can give greater probability of biological occupational injury [20, 21].

Hu et al. [22] examined a sample of 2014 frontline nurses working in two Wuhan hospitals, and more than half of the subjects reported moderate to high burnout. Weilenmann et al. [23] investigated the level of burnout in HCWs (857 physicians and 553 nurses) in Switzerland. The results showed high levels of anxiety, depression, and burnout symptoms. With small effects, women, nurses and other HCWs who had direct interaction with COVID-19 patients reported more symptoms than colleagues who did not.

Regardless of the effects of COVID-19, several studies have already indicated the effect of many psychological variables, such as depression, stress, anxiety, resilience, and satisfaction with life in burnout syndrome [24–30]. Thus, this issue deserves particular interest in the context of a pandemic. These studies took place at different points on the pandemic curve; however, no study has assessed the burnout and mental health status immediately after the suspension of the state of emergency. The “back-to-normal scenario” must be established under strict safety measures but give people the hope to rebuild and to return to life as close to normal as possible. The protection of HCWs should be a priority and policymakers should make evidence-based decisions [31, 32].

## Methods

### Aim

The aim of this study was to evaluate the relative contribution of socio-demographic and mental health variables on the three burnout dimensions of HCWs facing the COVID-19 pandemic.

### Study design

This cross-sectional quantitative web-based study examined HCWs living in Portugal. A survey was spread via social networks using a snowball technique and supported by health care institutions and professional organizations.

### Participants

The study population comprised HCWs in the Portuguese health system, including physicians, nurses, pharmacists, nutritionists, psychologists, other allied health professionals, and healthcare assistants. Of the 2008 responding participants, 1678 (83.6%) were women. The mean age of participants was 38 years old (SD = 10). Of the 945 (48.6%) participants who had children, 64% had children who were 12 years old or younger.

The residences of the participants were divided into seven regions based on the Portuguese Territorial Units for Statistics Level II (NUTS II): North (912 participants; 45.4%), Center (320 participants; 15.9%), Lisbon (418 participants; 20.8%), Alentejo (104 participants; 5.2%), Algarve (74 participants; 3.7%), Autonomous Region of Azores (56 participants; 2.8%), and Autonomous Region of Madeira (124 participants; 6.2%). Frontline HCWs were defined as those who indicating they worked face-to-face full time and part time.

### Procedures

Data collection took place from May 9th to June 8th, 2020. This period included a declaration of national calamity and easing of lockdown measures that followed a state of national emergency (between March 18th and May 2nd). A questionnaire built in Google® Forms platform was made available to participants via a link that was shared through direct e-mail and social networks.

Ethical procedures according to the Declaration of Helsinki were accomplished via analysis and approval of the study by the Ethics Committee of São João Hospital Center (Ref 184/2020 on May 7th, 2020). All participants gave informed consent online in compliance with General Data Protection Regulation guidelines for clinical research [33].

### Measures and covariates

Sociodemographic and other COVID-19-related background data were collected using a self-administered questionnaire. Psychological variables were collected using the Copenhagen Burnout Inventory (CBI); the Resilience Scale; the Depression, Anxiety, and Stress Scales (DASS-21); and the Satisfaction with Life Scale (SWLS). Burnout was measured by the validated Portuguese version of the CBI [18]. The CBI is a 19-item tool with three subscales: personal, work-related, and client-related burnout. The personal burnout subscale measures feelings of physical, emotional, and mental fatigue and exhaustion. The work-related burnout subscale assesses the symptoms that respondents’ attribute to work. The client-related burnout subscale describes feelings of physical and psychological fatigue and exhaustion that respondents attribute to their work with patients. All items are scored on a 5-point Likert scale. The score for each subscale is the average of item scores within the subscale and ranges from 0 to 100. Scores ≥50 in each of the three subscales were considered high-level burnout [18, 34]. These subscales are characterized by high internal consistency (original version: α = 0.84 and Portuguese version: α = 0.86, where α is the Cronbach’s alpha) [18, 34]. In the current study, α were 0.91, 0.89, and 0.89 for personal burnout, work-related burnout and client-related burnout, respectively.

The Resilience Scale [35] includes 25 items answered on a 7-point Likert scale ranging from strongly disagree (one) to strongly agree (seven). The Portuguese version presented high internal consistency, α = 0.89 [36]. In the current study, α = 0.95.

DASS-21 [37, 38] was used to evaluate mental health symptoms. This version consists of a 21-item 4-point Likert questionnaire that includes three self-reported subscales designed to measure the negative emotional states of depression, anxiety, and stress. Each of the three subscales contains seven items using a scale of zero (did not apply to me at all) to three (applied to me very much or most of the time). In the current study, α were 0.90, 0.84, and 0.90 for the depression, anxiety, and stress subscales, respectively.

SWLS is a 5-item 5-point Likert scale that assesses an individual’s global judgment regarding life satisfaction [39, 40]. The versions of this scale have acceptable or high internal consistency, original version: α = 0.87; Portuguese version: α = 0.77 [33, 34]. In this study, α = 0.86. In the Portuguese version, the scale has no cut-off point and has a possible range of 5 to 25 points.

### Data analysis

Data from Google® Forms were exported in a Microsoft Excel® 2016 spreadsheet, USA, and all statistical analyses were carried out using SPSS® Statistics (version 26.0; SPSS Inc., Chicago, Illinois, USA) and Jamovi 1.1.9.0. Absolute and relative frequencies, n (%) were used to describe categorical variables. Normally distributed quantitative variables were described by means and standard deviations (SDs). Non-normally distributed quantitative variables were described by medians (Med) and interquartile intervals [Q_1_; Q_3_] (although the means and standard deviations were also presented in these cases to facilitate comparison with other studies). Normality was verified by observing the histograms.

Differences between participants were analyzed using student t-tests for normally distributed quantitative data, Mann-Whitney U tests were used for quantitative non-normally distributed data, and chi-squared tests were used for categorical data. Pearson’s or Spearman’s coefficient was also used to explore the association between different domains (resilience, anxiety, depression, stress, life satisfaction, and burnout). The internal consistency of each of the subscales was assessed using Cronbach’s alpha (α), and a value above 0.7 was considered acceptable [41].

A separate multiple linear regression analysis was performed for each outcome (personal, work-related, and client-related burnout). The independent variables to include in each multiple regression were chosen by performing simple linear regressions with each variable in the dataset, including socio-demographics, variables related to COVID-19, and variables obtained from questionnaires (resilience, SWLS, and subscales from DASS-21). An additional file shows these results in more detail (see Additional file 1). All variables that correlated with the outcomes at *p* ≤ 0.05 in the simple regression were included in the multiple linear regression analyses. Only the significant variables were maintained in the final multivariate models for personal, work-related, and client-related burnout.

The results of linear regressions are presented with coefficient values (β), 95% confidence intervals (95% CIs), and *p*-values. The model was evaluated using the F statistic of the overall model test, p-values, and coefficients of determination (R^2^). The assumptions of the linear regression models were verified as follows: a) visual analysis of histogram to assess the normality of residuals; b) a t*-*test to determine whether mean residuals were equal to zero; and c) plots of residuals versus the fitted predictive values to check for homoscedasticity. Values of *p* ≤ 0.05 were considered significant.

## Results

### Sample characteristics

We received responses from 2061 respondents, but 53 respondents did not fully complete the questionnaires and were removed from the analysis. A total of 2008 HCWs completed the questionnaire.

The participants included 707 health technicians (35.2%), 511 physicians (25.4%), 409 nurses (20.4%), 88 pharmacists (4.4%), 83 psychologists (4.1%), 72 nutritionists (3.6%), 29 healthcare assistants (1.4%) and 21 workers in allied areas (1%). Among the participants, 157 (7.8%) worked in high-dependency units (intensive and intermediate care), 247 (12.3%) worked in emergency services, 485 (24.2%) worked in primary healthcare, 383 (19.1%) worked in inpatient areas and 167 (8.3%) worked in inpatient areas exclusively for COVID-19 patients.

Of all the participants, 524 (26.1%) reported having health problems:158 (30.2%) had a chronic respiratory disease and 119 (22.7%) had compromised immune systems. A total of 319 (15.9%) participants were caregivers of older people or with disabilities. The characteristics of the participants are summarized in Table 1.

**Table 1:** Sample characteristics of participants (*n* = 2008)

Characteristics	n	%
Marital status		
Single	780	38.8
Married/nonmarital partnership	1071	53.3
Divorced or Separated	141	7.0
Widowed	16	0.8
Parents		
Yes	975	48.6
No	1033	51.4
Lives with a person at risk for COVID-19 infection		
Yes	681	33.9
No	1327	66.1
Death of relative or friend during the pandemic period		
Yes	118	5.9
No	1890	94.1
Education level		
Graduate	1207	60.1
Postgraduate	801	39.9
Professional experience		
Five years or less	504	25.1
From 6 years to 15 years	745	37.1
More than 15 years	759	37.8
Frontline working position		
Yes	1398	69.7
No	609	30.3
Direct contact with infected people		
Yes	552	27.5
No	1456	72.5
Salary reduction		
Yes	710	35.4
No	1298	64.6
Diagnosed health problem		
Yes	524	26.1
No	1484	73.9
COVID-19 Tested		
Yes and, no but I’d like to do it	1487	74.1
No, I have no interest	521	25.9

### Results of levels of burnout dimensions and psychological variables

The average levels of burnout among HCWs were divided into low and high burnout groups. High levels of personal burnout were found in 1055 (52.5%) participants, and high work-related burnout was found in 1066 (53.1%). Resilience was moderate in 1021 (50.8%). Anxiety (66.9%), depression (70.6%) and stress (63.4%) were normal in most of participants (Table 2). The HCWs also showed high satisfaction with life with a median of 18 [14; 20] points.

**Table 2:** Descriptive statistics for burnout dimensions, resilience, anxiety, depression, and stress

Variable	n	%
Personal burnout		
Low level	953	47.5
High level	1055	52.5
Work-related burnout		
Low level	942	46.9
High level	1066	53.1
Client-related burnout		
Low level	1297	64.6
High level	711	35.4
Resilience		
Low	428	21.3
Moderate	1021	50.8
High	559	27.8
Anxiety		
Normal	1344	66.9
Mild	121	6.0
Moderate	302	15.0
Severe	101	5.0
Extremely severe	140	7.0
Depression		
Normal	1418	70.6
Mild	209	10.4
Moderate	228	11.4
Severe	85	4.2
Extremely severe	68	3.4
Stress		
Normal	1274	63.4
Mild	240	12.0
Moderate	250	12.5
Severe	171	8.5
Extremely severe	73	3.6

### Results of personal burnout, work-related burnout, and client-related burnout subscales: multivariate analysis

Socio-demographic, professional and psychological variables were identified as potential predictors according to the multiple linear regression (Table 3). Sex, parental status, marriage status and salary reduction were found to be significant factors for personal burnout. Women personal burnout levels were 4.51 points higher on average in comparison with men (*p* < 0.001). Having children under 12 years was associated with higher levels of personal burnout (β = 3.68; *p* < 0.001). Being diagnosed with health problems was associated with higher personal burnout (β = 1.84; *p* < 0.05) and work-related burnout (β = 1.68; *p* < 0.05). Single status was associated with significantly less personal burnout than marriage status/nonmarital partnerships (β = − 2.90, *p* < 0.001). Salary reduction was significantly associated with lower personal burnout levels (β = − 1.94, *p* < 0.05). Frontline working positions were associated with higher levels of personal burnout, work-related burnout, and patient-related burnout (β = 4.24, β = 3.91, and β = 2.35, *p* < 0.001, respectively).

**Table 3:** Regression coefficients for CBI subscales as outcomes and socio-demographic, professional, and emotional variables as predictors from univariate multiple linear regressions

Variables	Personal Burnout β [95% CI]	Work-related Burnout β [95% CI]	Client-related Burnout β [95% CI]
Gender			
Male	Reference		
Female	4.51 [2.71–6.31]***		
Marital status			
Married/nonmarital partnership	Reference		
Single	−2.90 [−4.52; −1.29]***		
Divorced or Separated	−2.46 [−5.18; 0.26]		
Widowed	−2.96 [−10.44; 4.52]		
Parental status			
No or older than 12 years old	Reference		
Yes, with 12 years old or less	3.68 [2.03; 5.33]***		
Education level			
Elementary or secondary school		Reference	
Graduate		3.91 [−0.11; 7.93]	
Postgraduate		6.28 [0.44; 12.13]*	
Master’s degree		5.76 [1.68; 9.84]**	
PhD		6.23 [0.69; 11.77]**	
Professional experience			
Five years or less			Reference
From 6 years to 15 years			4.46 [2.13; 6.78]***
More than 15 years			−0.80 [−3.12; 1.52]*
Frontline working position	4.24 [2.55; 5.93]***	3.91 [2.41; 5.43]***	2.35 [0.40; 4.31]*
Salary reduction	−1.94 [−3.50; −0.38]*		
Diagnosed health problem	1.84 [0.30; 3.38]*	1.68 [0.17; 3.19]*	
COVID-19 Tested			
Yes and, no but I’d like to do it	Reference		
No, I have no interest	−3.13 [−4.65; −1.61]***		
Direct contact with infected people	3. 27 [1.70; 4.83]***	3.45 [1.89; 5.00]***	
Death of relative or friend during pandemic period			−4.68 [−8.49; −0.87]*
Resilience	−0.08 [− 0.11; − 0.05]***	−0.05 [− 0.08; − 0.02]**	−0.07 [− 0.12; − 0.03]**
Satisfaction with life	−0.78 [− 0.98; − 0.58]***	−1.16 [− 1.35; − 0.96]***	−1.11 [− 1.38; − 0.85]***
Anxiety	0.33 [0.04; 0.62]***		
Depression	0.68 [0.41; 0.95]***	0.82 [0.56; 1.07]***	0.69 [0.34; 1.03]***
Stress	1.66 [1.41; 1.90]***	1.29 [1.09; 1.50]***	0.79 [0.51; 1.07]***
*R*^*2*^	0.475	0.407	0.187
*F*	120***	125***	57.5***

*Note*. *CI* confidence interval. *p ≤ 0.05; **p ≤ 0.01; ***p ≤ 0.001

HCWs who had direct contact with COVID-19 patients presented higher personal burnout levels (β = 3.27, *p* < 0.001) and work-related burnout levels (β = 3.45, *p* < 0.001). Higher levels of stress and depression in HCWs were significantly associated with higher levels of all burnout dimensions (*p* < 0.001). As presented in Table 3, higher levels of satisfaction with life and resilience were significantly associated with lower levels of all burnout dimensions.

## Discussion

Portuguese HCWs followed the trend of burnout seen in studies from other countries [23, 42]. Our findings show that more than half of HCWs experienced high levels of personal and work-related burnout, while most participants (64.6%) had low rates of client-related burnout. The results of psychological variables showed moderate resilience in 50.8% of the sample and normal levels for anxiety (66.9%), depression (70.6%) and stress (63.4%) in most of the participants. Notably, 74.9% of participants had six or more years of professional experience, which could contribute to a greater ability to manage anxiety and stress. Professional experience improves one’s clear awareness to solve problems, which can increase one’s confidence in professional actions, thus inducing less stress and anxiety.

The COVID-19 pandemic seems to have had an impact on the physical and psychological wellbeing of HCWs worldwide [5]. It is not unexpected that this new coronavirus has posed unprecedented challenges to HCWs. Previous research on burnout has already found that the highest prevalence rate of burnout occurs among HCWs in hospital emergencies [11]. Without comparing this situation to the pandemic, we emphasize that HCWs in hospital emergencies also deal with crisis situations. Thus, in a pandemic, exacerbation of this situation would be inevitable.

Emotional exhaustion related to low levels of mental health has been reported [7, 11], and effective interventions to support health care professionals are needed. Although the demands of medical practice may be a significant contributor to burnout, personal and family stressors may impose additional pressures. The COVID-19 pandemic has disrupted healthcare systems worldwide. A prolonged response period to the pandemic will lead to additional stress for HCWs, which will permeate further throughout the healthcare system [43].

Our findings reinforce the multidimensionality of the burnout syndrome. Indeed, each of the three burnout dimensions was associated with a specific set of covariates. Thus, consideration of these three dimensions is important when designing future burnout prevention programs for HCWs.

The contributions of socio-demographic and psychological variables on the three burnout dimensions were explored. We found that sex, marriage status, parental status, frontline worker positions, and direct contact with infected people significantly contributed to the outcomes. Our findings suggest that female sex is associated with higher levels of personal burnout, which is in line with previous research [44, 45]. These results might be explained by the double-workload role of women in society between their professions and home lives. These multiple responsibilities could result in a greater perception of personal burnout.

People who were single, widowed or divorced seemed to be were less susceptible to personal burnout than those who were married. This finding could be related to the dual role that married HCWs play, especially women, who were most of participants (83.6%) in this study. Such an association has been reported previously in a study on nurses [46].

HCWs with children under 12 years old were more likely to experience personal burnout than HCWs without children or children older than 12 years (during the period of data collection, the state allowed parents who had children up to the age of 12 years to work from home [47]. This was an interesting finding of the study since the roles of HCWs as parents and primary caregivers at home have rarely been investigated. With the spread of the coronavirus and the suspension of classes in schools, teleworking was encouraged. Parents have to juggle their roles as parents, workers, and many times, as teachers to help their children. Teleworking during the COVID-19 pandemic requires separating work and personal time, which could cause family obligations to intrude on work and work obligations to bleed into family time. This might lead teleworkers to work extra hours, resulting in burnout.

Working on the “frontline” is one of the few covariables that was significantly associated with all three dimensions of burnout. In a study conducted in China, the prevalence of burnout was high among frontline nurses [22]. The COVID-19 outbreak has led to a sharp increase in admissions and presentations to hospitals, which has impacted the workload of HCWs. Prior to this pandemic situation, these professionals were already considered as one of the groups most exposed to psychosomal risks [11]. The pandemic has exacerbated existing risks and triggered new risks, including risk of exposure to the pathogen, long working hours, increased volume and severity of patients, critical decision making, psychological distress, fatigue and the high concern that professionals could be potential vectors of disease transmission to their families.

Exposure to these risk factors can jeopardize the mental, physical, emotional, and social wellbeing of these professionals as well as the care process. It can also make it difficult for professionals to establish adequate therapeutic relationships. In the same direction, the significant determinants of personal and work-related burnout were health problems and directly participation in the diagnosis, treatment, and care for COVID-19 patients. A study carried out in Switzerland with HCWs demonstrated higher levels of burnout in the group that was in direct contact with patients [23].

Resilience, satisfaction with life, depression and stress were found to be potential predictors for all burnout dimensions, and anxiety was a potential predictor for personal burnout. The relationship between burnout and psychological dimensions has been documented in recent studies [26]. In this context, it can be argued that life satisfaction is a protector against developing burnout [30]. In this study, satisfaction with life seems to be a protective factor for burnout, which confirms previous research in this field.

A significant relationship was found between depression and all the dimensions of burnout, which highlights the importance of the problem and its prevention. Our findings are in line with the results from other studies [25, 26]. Several studies [24, 25] found consistent medium to high correlations between depression and burnout. According to our results, depression was identified as a potential predictor of burnout. Depression can have a negative impact on the health, performance, and productivity of workers, which can influence the quality of care provided and patients’ health [28]. To prevent negative impacts, coping strategies and resilience could have important roles [28]. In fact, according to our results, resilience was found to be a potential protective factor against burnout. In previous research, resilience was also found to be a protective factor for regulating and preventing burnout [27]. Resilience can be a psychological resource in performing emotional labor, and resilience-promotion programs should be implemented.

Stress also seems to be a risk factor for burnout. High levels of stress have serious consequences for the wellbeing of individuals and can lead to mental fatigue, difficulty in concentration, loss of immediate memory, and anxiety [48]. On the other hand, it could also empower an individual to deal with changing and adverse situations. Stress and burnout seem to be inseparable [29]. In a randomized controlled trial, Stier-Jarmer et al. [29] found that a program for stress reduction and burnout prevention was effective. The program aimed at reducing currently perceived stress, as well as providing strategies for dealing with stressors. The optimization of stress-management skills should be required.

Anxiety can be considered a reaction to threatening situations that acts as a protective factor [49]. However, if the anxiety is prolonged over the time it can result in suffering with an impact on the individual’s functioning [49]. According Spielberger [50], anxiety is divided in two types, trait anxiety and state anxiety. Trait anxiety is the individual proneness to anxiety, that is, the level how the person perceives stressful situations as threatening, and state anxiety is the reaction toward a situation after having judged it as threatening [50]. Anxiety seems to be a potential risk factor for personal burnout but not for work and client-related burnout. Personal burnout subscale measures physical and psychological fatigue, work-related burnout assesses the level of exhaustion and fatigue that derive from work, and client-related burnout analyze exhaustion because of the relation with clients [18]. Our findings suggest that exhaustion and fatigue does not derive from work or relation with the clients, but a consequence from physical and psychological fatigue. As stated, 69.7% of the study participants were frontline workers which required more personal effort and may have contributed to greater physical and psychological exhaustion. In addition, in the frontline, workers are faced with stressful situations and the level how the person perceives these situations as threatening can be higher. The literature in this field, in addition to advancing the existence of a correlation between anxiety and burnout, argues that this relationship is still unclear and further research is needed [26].

### Limitations

This study offered several interesting discoveries. However, some limitations should be considered. This study used a cross-sectional online survey, which might have limited the accessibility of people less familiar with the internet or less prone to using it. The sample was obtained by a snowball technique and might have not reached some classes or individuals. The study was carried out during a one-month period and is related to only a specific pandemic period, which corresponded to a relief of lockdown measures. In addition, has a transversal character and no data was analyzed before pandemic. No retrospective information was collected. Further investigation could ask if participants suffered of those symptoms before Covid-19 pandemic and if the symptoms increased during the pandemic.

Also, there could be a bias linked to socially desirable responding, that is, the tendency to reply to a questionnaire while giving a favorable image of oneself or to comply with the investigation goal. Future research could use the Social Desirability Scale, in association with the other tools used in this investigation, to improve the validity of questionnaire-based research.

## Conclusions

HCWs experience high burnout, which warrants attention and support from policy makers. Factors that potentially contribute to the level of burnout of HCWs include sex, marriage status, having children 12 years old or younger, education level, years of professional experience, frontline work, health problems and direct contact with infected people. It is essential to pay attention to the psychological wellbeing of these professionals. Occupational health surveillance can play an important role to improve HCWs wellbeing [51].

Measures could include the implementation of a recovery plan for these HCWs and the development of strategies for resilience training and self-care. Such efforts could increase the protective factors against environmental risks like those in the current pandemic, as well as develop positive factors for mental health. Also, is important, asking for help from other professionals ensures different perspectives. The more experience a professional has the greater is the probability he or she will simplify information. Intervention groups and supervision to discuss their interpretations and proposals for intervention with colleagues can be important and may act as a protective measure in several ways, both in professional practice and in promoting psychological well-being.

## Supplementary Information

**Additional file 1:.** Factors associated with personal, work, and client-related burnout identified by simple linear regression. The independent variables to include in each multiple regression were chosen by performing simple linear regressions with each variable in the dataset.

## Data Availability

The datasets during or analyzed during the current study are available from the corresponding author on reasonable request.

## Abbreviations

HCWs: Healthcare workers
SARS: Severe Acute Respiratory Syndrome
MERS: Middle East Respiratory Syndrome
SD: Standard deviation
NUTS II: Portuguese Territorial Units for Statistical Level II
Ref: Reference
CBI: Copenhagen Burnout Inventory
DASS-21: Depression, Anxiety, and Stress Scales
SWLS: Satisfaction with Life Scale
α: Cronbach’s alfa
USA: United States of America
SPSS: Statistical Package for the Social Sciences
n: Absolute frequencies
SD: Standard deviation
Med: Medians
Q1: First quartile
Q3: Third quartile
P: *p*-value
β: Coefficient value
CIs: Confidence intervals
R^2^: Coefficient of determination
