# Supplementary material for: Burnout among Portuguese healthcare workers during the COVID-19 pandemic
Source: BMC Public Health. 2020 Dec 7;20:1885. doi: 10.1186/s12889-020-09980-z (PMC7720923; doi:10.1186/s12889-020-09980-z)
Supplement: Supplementary file 1 — Additional file 1:. Factors associated with personal, work, and client-related burnout identified by simple linear regression. The independent variables to include in each multiple regression were chosen by performing simple linear regressions with each variable in the dataset. [file 12889_2020_9980_MOESM1_ESM.docx]

Additional File 1. Factors associated with personal, work, and client-related burnout identified by simple linear regression

| Variable | Personal Burnout | Work-related Burnout | Client-related Burnout |
| --- | --- | --- | --- |
|  | β (95% CI) | | |
| Sex |  |  |  |
| Male | Reference | Reference | Reference |
| Female | 7.77*** [5.34; 10.20] | 3.72*** [1.43; 6.01] | -0.67 [-3.35; 2.01] |
| Marriage status |  |  |  |
| Married/nonmarital partnership | Reference | Reference | Reference |
| Unmarried | -2.82** [-4.72; -0.91] | -1.44 [-3.23;0.34] | 0.30 [-1.78; 2.39] |
| Divorced or Separated | 0.84 [-2.78; 4.46] | 1.48 [-1.92; 4.87] | -0.57 [-4.54; 3.40] |
| Widowed | -8.24 [-18.42; 1.94] | -8.07 [-17.61; 1.48] | -8.66 [-19.83; 2.50] |
| Have children |  |  |  |
| No or older than 12 years old | Reference | Reference | Reference |
| Yes, with 12 years old or less | 3.27*** [1.32; 5.22] | 1.24 [-0.59; 3.07] | 1.35 [-0.78; 3.49] |
| Education level |  |  |  |
| Elementary or secondary school | Reference | Reference | Reference |
| Graduate | 0.21 [-5.33; 5.76] | 5.96* [0.78; 11.10] | 2.19 [-3.87; 8.25] |
| Postgraduate | 1.50 [-6.57; 9.56] | 9.05* [1.51; 16.60] | 5.88 [-2.93; 14.70] |
| Master’s degree | 0.77 [-4.86; 6.40] | 7.70** [2.45; 13.00] | 3.81 [-2.34; 9.97] |
| PhD | -1.88 [-9.51; 5.75] | 4.43 [-2.69; 11.60] | -2.54 [-10.87; 5.80] |
| Caretaker |  |  |  |
| No | Reference | Reference | Reference |
| Yes | 2.74* [0.27; 5.21] | 2.63* [0.32; 4.95] | -1.25 [-3.96; 1.46] |
| Lives with a risk person for COVID-19 |  |  |  |
| No | Reference | Reference | Reference |
| Yes | 1.91* [0.00; 3.81] | 1.08 [-0.71; 2.87] | 0.23 [-1.86; 2.32] |
| Death of relative or friend during the pandemic |  |  |  |
| No | Reference | Reference | Reference |
| Yes | -0.26 [-4.11; 3.58] | -0.77 [-4.37; 2.83] | -4.20* [-8.41; 0.00] |
| Years of professional experience |  |  |  |
| Five years or less | Reference | Reference | Reference |
| From 6 years to 15 years | 1.88 [-0.46; 4.21] | 2.14 [-0.05; 4.32] | 2.67* [0.12; 5.22] |
| More than 15 years | 0.71 [-1.62; 3.04] | 1.15 [-1.03; 3.33] | -1.88 [-4.42; 0.65] |
| Frontline working position |  |  |  |
| No | Reference | Reference | Reference |
| Yes | 7.26*** [5.32; 9.20] | 5.39*** [3.57; 7.22] | 2.45* [0.30; 4.60] |
| Salary reduction |  |  |  |
| No | Reference | Reference | Reference |
| Yes | -3.64*** [-5.53; -1.76] | -2.52** [-4.29; -0.75] | -0.06 [-2.13; 2.01] |
| Diagnosed health problem |  |  |  |
| No | Reference | Reference | Reference |
| Yes | 6.30*** [4.26; 8.34] | 5.65*** [3.74; 7.56] | 1.48 [-0.77; 3.73] |
| COVID-19 Tested |  |  |  |
| Yes and, no but I'd like to do it | Reference | Reference | Reference |
| No, I have no interest | -6.20*** [-8.24; -4.15] | -3.75*** [-5.68; -1.83] | -0.63 [-2.89; 1.63] |
| Direct contact with infected people |  |  |  |
| No | Reference | Reference | Reference |
| Yes | 7.21*** [5.21; 9.22] | 6.36*** [4.48; 8.23] | 6.36*** [4.48; 8.23] |
| Resilience | -0.31*** [-0.35; -0.27] | -0.27*** [-0.31; -0.24] | -0.24*** [-0.28; -0.20] |
| Anxiety | 2.89*** [2.68; 3.10] | 2.41*** [2.21; 2.62] | 1.80*** [1.54; 2.06] |
| Depression | 2.81*** [2.62; 3.00] | 2.56*** [2.38; 2.74] | 1.96*** [1.73; 2.20] |
| Stress  Satisfaction with life | 2.69*** [2.54; 2.85]  -1.95*** [-2.16; -1.73] | 2.25*** [2.10; 2.40]  -2.14*** [-2.34; -1.94] | 1.63*** [1.43; 1.83]  -1.84*** [-2.08; -1.60] |

**p* ≤ 0.05; ***p* ≤ 0.01; ****p* ≤ 0.001
